# Novel Sodium Channel Inhibitor From Leeches

**DOI:** 10.3389/fphar.2018.00186

**Published:** 2018-03-06

**Authors:** Gan Wang, Chengbo Long, Weihui Liu, Cheng Xu, Min Zhang, Qiong Li, Qiumin Lu, Ping Meng, Dongsheng Li, Mingqiang Rong, Zhaohui Sun, Xiaodong Luo, Ren Lai

**Affiliations:** ^1^Key Laboratory of Bioactive Peptides of Yunnan Province/Key Laboratory of Animal Models and Human Disease Mechanisms of Chinese Academy of Sciences, Kunming Institute of Zoology, Kunming, China; ^2^Graduate School of University of Chinese Academy of Sciences, Beijing, China; ^3^State Key Laboratory of Phytochemistry and Plant Resources in West China, Kunming Institute of Botany, Chinese Academy of Sciences, Kunming, China; ^4^Sino-African Joint Research Center, Chinese Academy of Sciences, Wuhan, China; ^5^Department of Clinical Laboratory, Guangzhou General Hospital of Guangzhou Military Command of PLA, Guangzhou, China; ^6^Life Sciences College of Nanjing Agricultural University, Nanjing, China

**Keywords:** leech therapy, blood-sucking, sodium channel, pain, analgesia

## Abstract

Considering blood-sucking habits of leeches from surviving strategy of view, it can be hypothesized that leech saliva has analgesia or anesthesia functions for leeches to stay undetected by the host. However, no specific substance with analgesic function has been reported from leech saliva although clinical applications strongly indicated that leech therapy produces a strong and long lasting pain-reducing effect. Herein, a novel family of small peptides (HSTXs) including 11 members which show low similarity with known peptides was identified from salivary glands of the leech *Haemadipsa sylvestris*. A typical HSTX is composed of 22–25 amino acid residues including four half-cysteines, forming two intra-molecular disulfide bridges, and an amidated C-terminus. HSTX-I exerts significant analgesic function by specifically inhibiting voltage-gated sodium (Na_V_) channels (Na_V_1.8 and Na_V_1.9) which contribute to action potential electrogenesis in neurons and potential targets to develop analgesics. This study reveals that sodium channel inhibitors are analgesic substances in the leech. HSTXs are excellent candidates or templates for development of analgesics.

## Introduction

As specific bloodsucking ectoparasites, leeches have to penetrate hosts’ body surface and suppress the reactions of hosts to injuries, such as swelling, pain, and inflammation to remain undetected and successfully get blood meal, suggesting that anesthetic agents may delivered during the blood-sucking process. Furthermore, they have to overcome hemostatic and vasoconstriction reactions in hosts to ensure a steady and sustained blood flow to the feeding site. Leeches inject saliva into hosts to counteract the reactions of hosts.

With many clinically challenges, pain affects millions of individuals worldwide and remains poorly understood. VGSCs (voltage-gated sodium channels) are essential for pain perception because they play a critical role in the generation and propagation of action potentials in excitable cells, including neurons, muscle cells, and cardiomyocytes. Nine subtypes (Na_V_1.1–1.9) of VGSCs have been identified in mammals. VGSCs have been broadly classified as TTX-S (TTX-sensitive) and TTX-R (TTX-resistant) by their sensitivities to tetrodotoxin (TTX). The majority of DRG (dorsal root ganglion) neurons express at least six Na_V_ subtypes (Cummins et al., 1999; Waxman et al., 1999): Na_V_1.1, Na_V_1.6, Na_V_1.7, Na_V_1.8, Na_V_1.9, and Nax (Dib-Hajj et al., 1998). The TTX-S Na_V_ current produced by Na_V_1.1, Na_V_1.6 and Na_V_1.7 is fast-inactive. Whereas, Na_V_1.8 and Na_V_1.9 produce the slow-inactivating TTX-R current (Akopian et al., 1996).

Different sodium channel subtypes have specific distributions and functions. Sodium channel Na_V_1.8 is mainly expressed in peripheral neurons, in particular within retinal amacrine and ganglion cells (O′Brien et al., 2008), small and medium-sized DRG neurons, and nociceptive afferent fibers (Benn et al., 2001; Shields et al., 2012); Na_V_1.9 is expressed more widely. It is found in the hippocampus, cortex (Blum et al., 2002), photoreceptors and Müller glia, small diameter DRG, trigeminal ganglia, and the intrinsic sensory neurons of the gut (Fang et al., 2002; Rugiero et al., 2003; Padilla et al., 2007).

Compared with other TTX-S channels, Na_V_1.8 is known to produce a slow-inactivating TTX-R current and is characterized by significantly depolarized activation and inactivation. Na_V_1.9 exhibits unique biophysical properties that include a hyperpolarized voltage-dependent activation and inactivation curves that overlap to produce a large ‘window current’ and very slow activation and inactivation kinetics (Dib-Hajj et al., 2002). Studies in knockout animals have clearly established a role of Na_V_1.8 in pain (Akopian et al., 1996; Zimmermann et al., 2007). Furthermore, gain-of-function mutations of Na_V_1.8 have been found in human subjects with painful neuropathies. These mutations in Na_V_1.8 channel markedly alter the excitability of DRG neurons (Faber et al., 2012; Huang et al., 2013; Han et al., 2014). Similarly, several monogenic human pain disorders are linked to altered Na_V_1.9 channel function. For example, some mutations in SCN11A (Na_V_1.9) resulted in a gain of function at the channel level and were associated with increased pain (Zhang et al., 2013; Huang et al., 2014, 2017), whereas in other study, a mutation (L811P) at the Na_V_1.9 channel resulted in an inability to experience pain (Leipold et al., 2013). In addition, studies using prostaglandin E2, protein kinase C, intracellular GTPγS (hydrolysis-resistant GTP analog), or G proteins have demonstrated a link between inflammatory pathways and Na_V_1.9 channel (Baker et al., 2003; Rush and Waxman, 2004; Baker, 2005; Ostman et al., 2008; Ritter et al., 2009). Na_V_1.9 is important for the perception of pain in response to cold. Cold pain sensation was greatly reduced in neurons from Na_V_1.9 knockout mice (Leipold et al., 2015; Lolignier et al., 2015). These observations indicate that Na_V_1.8/9 represents a high-valued target for development of analgesics.

Blood-sucking leeches have been used for medical purposes in humans for more than 2000 years. Leeches have been approved as medicinal materials or medical device in many countries (Hyson, 2005). As a traditional anti-thrombosis medicinal material, leeches have been used in China for over 1000 years. The FDA has approved leeches as a medical device for plastic and reconstructive surgery in July 2004 (Rados, 2004). Recently, leech therapy has been approved as legal therapeutic intervention in Europe. Especially, extensive attention has been paid for medicinal leech therapy in pain syndromes, such as osteoarthritis, epicondylitis, vertebrogenic pain syndromes/lower back pain, hematoma/swelling/edema/contusion/distortion, varicose veins/leg ulcer/phlebitis/thrombophlebitis, and cancer pain (Koeppen et al., 2014). Analgesic effect of leech therapy has been approved in many clinic trials. The analgesic effect from leech therapy is rapid, effective, and long-lasting in many conditions (Singh, 2010). However, no specific substance with analgesic function has been reported from leech yet. In this study, we identified a novel group of peptides targeting Na_V_1.8 and Na_V_1.9 to produce analgesic effect for the first time.

## Materials and Methods

### Collection of Saliva Sample From *H. sylvestris*

*Haemadipsa sylvestris* (total weight 500 g) was collected from jungles in Yunnan, China. Saliva sample was collected as described below. Living leeches were frozen quickly in liquid nitrogen. Leech saliva located in the subcutaneous of pharynx was separated, collected and triturated by grinder (Joyoung, China) at low temperature. Triturated saliva sample was dissolved in deionized water, and immediately centrifuged to remove debris. The supernatant [crude extract of saliva (CES)] was lyophilized and kept at -20°C till use. All of the experimental protocols using animals were approved by the Animal Care and Use Committee at Kunming Institute of Zoology, Chinese Academy of Sciences (SYDW-2013018).

### Peptide Purification

The CES was applied to a Sephadex G-50 gel filtration column (GE Healthcare, 20–100 μm, 2.6 cm × 100 cm) which was equilibrated with Tris-HCl buffer (50 mM Tris-HCl, pH 8.9). Sample fractionation was performed by eluting the column using Tris-HCl buffer. Each eluted fraction with a volume of 3.0 ml was collected every 10 min and the absorbance of the eluate was measured at 215 and 280 nm. The effects of eluted fractions (desalted) on VGSCs were assayed using whole-cell patch clamp techniques as described below. The fractions containing VGSCs-inhibiting activity were pooled, lyophilized, and resuspended in 10 ml of the Tris-HCl buffer. The fractions were further purified by using RP-HPLC with C18 column (SunFire^TM^ Prep C_18_, 5 μm, 250 mm × 10 mm, Waters) by using acetonitrile containing 0.1% trifluoroacetic acid (TFA) as elution solvent. The absorbance at 215 and 280 nm was monitored by UV detector (Waters 2489).

### Determination of Amino Acid Sequence

Edman degradation was used to analyze the amino sequence of purified peptides by pulsed liquid-phase sequencer (Applied Biosystems, model 491). Molecular weight was determined by MALDI-TOF-MS (Matrix-assisted laser desorption ionization time-of-flight mass spectrometry, autoflex^TM^ speed, Bruker) by using positive reflector mode and reported as the monoisotopic [M + H]^+^ ions, with ± 0.001% accuracy of mass determinations.

### Construction and Screening of *H. sylvestris* Salivary Gland cDNA Library

Total RNA was extracted using TRIzol Reagent (Invitrogen, United States) from salivary gland (100 mg) of *H. sylvestris* according to our previous methods (An et al., 2013). An Oligotex mRNA Mini kit (Qiagen, Duesseldorf, Germany) was used to extract mRNA. cDNA library was constructed by using a Creator SMART^TM^ cDNA Library Construction Kit (Clontech, Palo Alto, CA, United States) following manufacturer’s instructions, and finally a library of about 2 × 10^6^ independent colonies was produced.

Primers HS1 (5′-GGCAATATTCAAGTCGAAGCTTGC-3′) designed according to the homologous sequences determined by transcriptome sequencing, and CDS III (5′-ATTCTAGAGGCCGAGGCGGCCGACATG-3′) were used for 3′ end DNA amplification. Primers HS2 (5′-GAAACATTTTAATTTTTAGAGGTTCATCC-3′) designed according to transcriptome data, and CDSIII were used for 5′ end DNA amplification. The PCRs were performed by using Advantage polymerase (Clontech, Palo Alto, CA, United States) as follows: 5 min at 95°C followed by 35 cycles of 10 s at 95°C, 30 s at 50°C, 40 s at 72°C, and last 10 min at 72°C. The PCR products were purified by DNA Gel Extraction Kit (Tiangen, China) and ligated into pMD19-T vector (TaKaRa Biotechnology Dalian, Co., Ltd., China) following manufacturer’s instructions. DNA sequencing was performed on an Applied Biosystems DNA sequencer (ABI PRISM377).

### Intra-molecular Disulfide Bridge Analysis

After incubation in 6 M guanidine solution (pH 3.0) at 37°C for 30 min, native peptide (3 mg) was partially reduced with Tris (2-carboxyethyl) phosphine hydrochloride (0.1 M) at 37°C for 40 min in 0.1 M citrate buffer solution (pH 3.0). The products were purified and lyophilized. The reduced half-cysteines in the peptide were alkylated with 0.5 M iodoacetamide in 0.5 M *N*-methyl morpholine solution (pH 8.3) at 25°C for 30 s. Amino acid sequence of reduced peptide was analyzed by Edman degradation (Wu et al., 1996).

### Structure Prediction and Modeling

The structure of HSTX-I was modeled (Supplementary Data Sheet 2) by homology modeling using 3BT4 (PDB ID) as a template. The suitable template for modeling was selected by BLAST result (Supplementary Figure 6) and disulfide bond assignment (Supplementary Figure 2). After suitable template is selected, the PDB file is parsed to extract its sequence. The amino acids of the template are replaced with that of HSTX-I to achieve template sequence alignment. Minimization (QM-MM) protocol was used to minimize the energy of HSTX-I sidechains through geometry optimization using the hybrid delocalized internal coordinate (HDLC) optimizer. The QM region of atoms is treated by a quantum calculation using the DMol^3^ server and the rest MM is handled by the CHARMm forcefield.

The structure was predicted and modeled using the Discovery Studio (3.1, BIOVIA, San Diego, CA, United States) software and following the data from Edman degradation.

### Tissue Distribution of the Analgesic Peptide by qPCR

RNA was extracted from head, body, and tail of *H. sylvestris*, respectively. The expression pattern analyzed by qPCR was repeated three times. HSTX-I and GAPDH primers were designed using IDT Primerquest tools^1^ as listed in Supplementary Table 1. SYBR Green PCR kit (Applied Biosystems) was used to perform qPCR. The qPCR procedures were as follows: 5 min of denaturation at 95°C, followed by 40 cycles of amplification with 5 s of denaturation at 94°C, 20 s of annealing according to the melting temperatures provided in Supplementary Table 1 and 15 s of extension at 72°C. The fluorescence data were collected at the end of each cycle. After a final extension at 72°C for 10 min, the specificity of the amplified product was evaluated by melting curve.

### Voltage-Clamp and Current-Clamp Recordings

Animal studies followed a protocol (SYDW-2013018) approved by the Animal Care and Use Committee at Kunming Institute of Zoology, Chinese Academy of Sciences. For patch clamp recording, DRG neurons were isolated, as previously reported (Yang et al., 2013). Briefly, rat DRG neurons were harvested and incubated at 37°C for acute dissociation in enzymatic solution [DMEM medium (Corning, NY, United States) with 0.3% collagenase (Sigma, St. Louis, MO, United States) and 0.7% trypsin (Sigma, St. Louis, MO, United States)]. After 20 min, the enzymatic solution was replaced by DMEM complete growth medium with 0.3% trypsin inhibitor (Sigma, St. Louis, MO, United States) and maintained in short-term primary culture. All cells were used within 12 h of isolation.

Ca^2+^, K^+^, and Na^+^ currents were recorded from cells using the whole-cell patch clamp technique performed as previously described (Intlekofer et al., 2013; Leipold et al., 2015; Lolignier et al., 2015; Xu et al., 2015). The P/4 protocol was used to subtract linear capacitive and leakage currents. For sodium channel current recordings on DRG neurons, the bath solution contained the following (in mM): 30 NaCl, 25 D-glucose, 1 MgCl_2_, 1.8 CaCl_2_, 90 TEA-Cl, 5 CsCl, and 5 HEPES at pH 7.4; the pipettes internal solution contained (in mM): 135 CsF, 10 NaCl, and 5 HEPES at pH 7.4. Adding 300 nM TTX (tetrodotoxin) and neurons diameter (<25 μm) were used for discriminating TTX-R from TTX-S Na_V_ channels. Cells were activated by a 100-ms step depolarization to -10 mV from a holding potential of -80 mV for Na_V_ currents. For potassium channel, the external solution was (in mM): 130 NaCl, 5 KOH, 12 D-glucose, 2 CaCl_2_, 2 MgCl_2_, and 10 HEPES, pH 7.2; pipettes were filled with a solution containing (in mM): 120 KF, 20 NMDG (*N*-methyl-D-glucamine), 11 EGTA, 2 Na-ATP, 0.5 GTP, and 10 HEPES, pH adjusted to 7.2 with KOH. Cells were evoked by a 500-ms depolarizing potential of +10 mV from a holding potential of -80 mV to record K_V_ currents. For calcium current recording, the pipette internal solution contained the following (in mM): 110 CsCl, 5 MgCl_2_, 10 EGTA, 25 HEPES, and 2 Mg-ATP, pH adjusted to 7.2 with CsOH; the bath solution contained the following (in mM): 125 TEA-Cl, 10 BaCl_2_, 5 HEPES, 5 D-glucose, pH adjusted to 7.3 with CsOH. Cells were activated by a 150-ms step depolarization to +10 mV from a holding potential of -90 mV for Ca_V_ currents. ∼1 μM TTX was added in potassium and calcium bath solution to block Na_V_ current. For Na_V_1.8 channel voltage-clamp recording, DRG neurons (diameter < 25 μm with 300 nM TTX) were held at -70 mV to inactivate Na_V_1.9 channels. Furthermore, neurons with potential contamination of Na_V_1.9 current were excluded. For Na_V_1.9 channels voltage-clamp recording, Na_V_1.8-null mouse DRG neurons (diameter < 20 μm with 300 nM TTX) were activated by a 100-ms step depolarization to -40 mV from a holding potential of -110 mV for Na_V_1.9 currents. The bath and pipettes solution for Na_V_1.8 and Na_V_1.9 contained the following (in mM): 150 NaCl, 2 KCl, 5 D-glucose, 1 MgCl_2_, 1.5 CaCl_2_, and 10 HEPES at pH 7.3; the pipettes internal solution contained (in mM): 105 CsF, 35 NaCl, 10 HEPES, 10 MgCl_2_, and 10 EGTA at pH 7.3.

Concentration-response curves were fitted using the following Variable slope model: Y = Bottom + (Top-Bottom)/(1+10^∧^((LogIC_50_-X)^∗^HillSlope)) where HillSlope is the steepness of the family of curves, Top and Bottom are the fraction of current resistant to inhibition at low toxin concentration and high toxin concentration, respectively. For current–voltage (I–V) relationships, cells were held at -70 mV (-110 mV for Na_V_1.9 channels) and depolarized to potentials from -80 mV (-100 mV for Na_V_1.9 channels) to +40 mV in 5 mV increments for 100 ms, and peak currents were recorded. Conductance–voltage (G–V) relationships were determined from peak current (I) versus voltage relationships as G = I/(V-V_rev_), where V was the test potential, and V_rev_ was the extrapolated reversal potential. Steady-state fast inactivation was achieved with a series of 500-ms prepulses -100 mV (-120 mV for Na_V_1.9) to +10 mV in 10 mV increments, and the remaining non-inactivated channels were activated by a 40-ms step depolarization to 0 mV (-40 mV for Na_V_1.9). Steady-state inactivation curves were fitted using the Boltzmann equation. The patch pipettes with DC resistances of 2–3 M were fabricated from borosilicate glass tubing (VWR micropipettes, 100 mL, VWR, Radnor, PA, United States) using a two-stage vertical microelectrode puller (PC-10, Narishige, Tokyo, Japan) and fire-polished by a heater (Narishige, Tokyo, Japan). Recordings were sampled at a rate of 50 kHz, and filtered at 3 kHz.

Before proceeding to current-clamp recordings, the same amplifier with the same configurations as voltage-clamp recordings was used. Pipette solution and bath solution was the same as previously described. The voltage threshold was determined by the first action potential elicited by a series of 1000 ms depolarizing current injections that increased in 50-pA increments. Cells with unstable (>10% variation) resting membrane and action potential overshoot of <40 mV were excluded for data collection.

All data recordings were performed at room temperature using an Axon Multiclamp 700B amplifier (Molecular Devices, Silicon Valley, CA, United States).

### Anti-nociceptive Test

To evaluate anti-nociceptive function of samples on mice (female, 18–22 g), several animal models including formalin-induced paw licking, abdominal writhing induced by acetic acid, and hot plate test were used according to previous methods (Yam et al., 2008). Morphine and saline were used as positive and negative control, respectively.

In formalin-induced paw licking model, mice were injected intraperitoneally with test sample dissolved in 100 μl of saline. Thirty minutes later, animals were injected with 20 μl of 1% (vol/vol) formalin at the subcutaneous tissue on plantar surface of right hind paw. Mice were then placed individually into open polyvinyl cages (20 cm × 40 cm × 15 cm). Times of licking the injected paw were recorded by digital video camera during phase I (0–5 min post-injection) and II (15–30 min post-injection).

In the model of abdominal writhing induced by acetic acid, after 30 min of intraperitoneal injection of test sample in one side of abdomen of mice, 100 μl of 1.5% (vol/vol) acetic acid was injected into the other side of abdomen. Mice were placed into open polyvinyl cages (20 cm × 40 cm × 15 cm) immediately, and nociceptive behaviors were counted cumulatively over a period of 30 min.

For hot plate test, mice (female) were placed on a hot plate (ZH-Z organ measurement system, China) with temperature maintained at 55 ± 0.5°C. The response time between placement of the mice on the plate and licking the paws or jumping was recorded as latency (in seconds). The latency was recorded at 0, 30, 60, 90, and 180 min after the intraperitoneal administration of test sample dissolved in 0.9% saline (Yam et al., 2008).

### Data Analysis

Electrophysiological data were analyzed using program Clampfit 10.0 (Molecular Devices) and GraphPad Prism 6. Statistical significance was determined by paired Student’s *t*-tests or ANOVA. Other experimental results are expressed as means ± SEM. ^∗^*P* < 0.05 and ^∗∗^*P* < 0.01 are significantly different compared with the control.

### Ethics and Biosecurity Statement

All of the experimental protocols using animals were approved by the Animal Care and Use Committee at Kunming Institute of Zoology, Chinese Academy of Sciences (SYDW-2013018). All the researches involving biohazards, biological select agents, toxins, restricted reagents and venom were approved by the Biosafety Committee of Kunming Institute of Zoology, Chinese Academy of Sciences (KZR-2015047).

## Results

### Purification of Peptide Possessed Activity to Inhibit VGSCs (Voltage-Gated Sodium Channels)

As illustrated in Supplementary Figure 1A, the extracts of the leech salivary glands were divided into six fractions by Sephadex G-50 gel filtration. Fraction III was found to inhibit TTX-R (tetrodotoxin-resistant) sodium channel currents and was purified further by a C_18_ RP-HPLC (reverse-phase high performance liquid chromatography) (Supplementary Figure 1B). A peptide named HSTX-I, which contained activity to inhibit TTX-R sodium channel currents was purified.

### Amino Acid Sequence of HSTX-I

**FIGURE 1:**
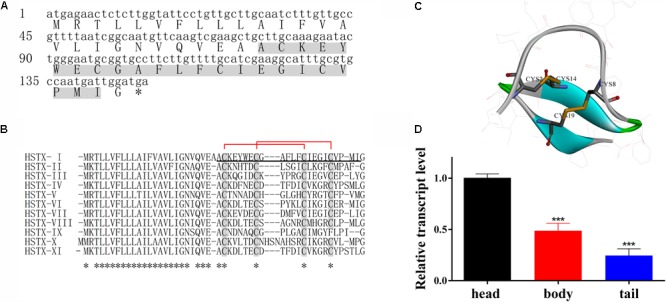
HSTX-I from *Haemadipsa sylvestris* saliva. **(A)** cDNA sequence encoding HSTX-I precursor. Mature peptide was determined by Edman sequencing (gray background). Asterisks indicate stop codon. **(B)** Sequence alignment of HSTX-I with its homologous sequences identified from cDNA library of *H. sylvestris* salivary glands. The conserved half-cysteines are highlighted by gray background. Asterisks indicate conserved residues. The mature peptide is underlined. The assignment of the disulfide bonds of HSTX-I is Cys2-Cys14 and Cys8-Cys19 which are indicated by red lines. **(C)** The secondary structure and disulfide bonds of HSTX-I are demonstrated by Discovery Studio. The disulfide bonds are linked in yellow line, the turn is shown in green, beta strands are shown in blue and random coils are shown in gray. **(D)** Tissue distribution of HSTX-I revealed by qPCR in *H. sylvestris*. Statistically significant differences compared with the head group (calculated using a two-tail Student’s *t*-test) are indicated by ^∗∗∗^*P* < 0.001.

The complete amino sequence (ACKEYWECGAFLFCIEGICVPMI) of HSTX-I was determined by Automated Edman Degradation. The cDNA sequence encoding the precursor of HSTX-I was cloned from the cDNA library of *H. sylvestris* salivary gland (**Figure 1A**). The precursor is composed of 49 AA (amino acid) residues including a predicted 25-AA signal peptide, a mature HSTX-I and a glycine at the C-terminal, which provides a -NH_2_ for- C-terminal amidation like other neuropeptides or antimicrobial peptides (Hosseini et al., 2013; Dennison et al., 2015).

Ten homologous sequences of HSTX-I were also cloned from the cDNA library (**Figure 1B**). All of them share highly conserved cysteine motifs and signal sequence. BLAST search did not find any similarity to other known peptides or proteins, indicating these peptides are a novel peptide family. Eleven members of this peptide family are found in the cDNA library of the leech salivary glands, which confirms the diversity of the peptide family in this species.

The mature peptide contains four cysteines, which form two intra-molecular disulfide bonds, Cys2-Cys14 and Cys8-Cys19 (**Figures 1B,C** also see Supplementary Figure 2A). Carboxypeptidase Y treatment did not release the C-terminal amino acid residue, indicating that the C-terminal residue in HSTX-I is amidated. MALDI-TOF mass spectrometry (Supplementary Figure 2B) analysis gave an observed molecular mass of 2621.14 Da which is identical with the calculated molecular mass (2621.17 Da) of HSTX-I with two intra-molecular disulfide bridges and an amidated C-terminus.

The distribution of HSTX-I in leech was evaluated by qPCR (real-time quantitative PCR). The results showed that the expression level of HSTX-I in the head of leech was 2–3 times higher than those in body and tail (**Figure 1D**).

### Effects of HSTX-I on Na_V_1.8/1.9 Channels

**FIGURE 2:**
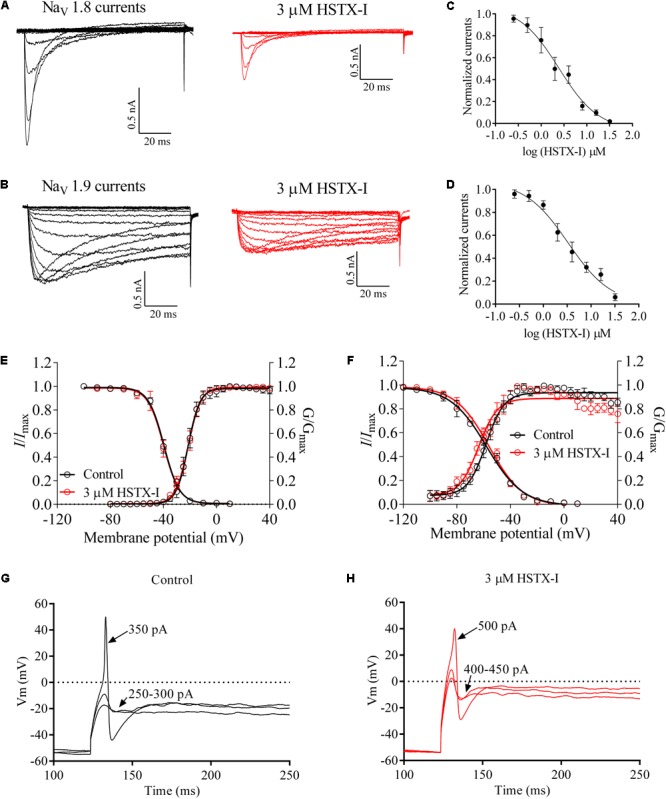
HSTX-I inhibited Na_V_1.8/1.9 channels (also see Supplementary Figure 5). **(A)** Representative current traces of rNa_V_1.8 recorded from the rat small DRG neurons (<25 μm) with 300 nM TTX at holding voltage of –70 mV. **(B)** Representative current traces of mNa_V_1.9, recorded from the Na_V_1.8-null mouse small DRG neurons (diameter < 25 μm with 300 nM TTX) at holding potential of –110 mV. Control currents are shown in black and the inhibition of rNa_V_1.8 **(A)** and mNa_V_1.9 **(B)** by the indicated concentrations of HSTX-I are shown in red. **(C)** Concentration-response curves for the inhibition of rNa_V_1.8 by HSTX-I. Cells were activated by a 100-ms step depolarization to –10 mV from a holding potential of –70 mV for the IC_50_ of HSTX-I on rNa_V_1.8 channel. **(D)** Concentration-response curves for the inhibition of mNa_V_1.9 by HSTX-I. Cells were activated by a 100-ms step depolarization to –40 mV from a holding potential of –110 mV for the IC_50_ of HSTX-I on mNa_V_1.9 channel. Data are represented as mean ± SEM. **(E)** Effect of 3 μM HSTX-I on the conductance–voltage (G–V) relationship (right) and voltage dependence of steady-state inactivation (left) of rNa_V_1.8 channels. **(F)** HSTX-I (3 μM) shifted the G–V relationship of mNa_V_1.9 approximately 5 mV in a negative direction, but it did not induce a shift in steady-state inactivation. **(G)** Responses of a small DRG neuron to the sub-threshold (250–300 pA) and the threshold depolarizing current injections (350 pA). **(H)** Responses of a small DRG neuron treated with 3 μM HSTX-I to the sub-threshold (400–450 pA) and the threshold depolarizing current injections (500 pA). Arrows with numbers indicate the current amplitudes used to elicit the responses.

Because HSTX-I showed inhibitory effects on TTX-R sodium channels (see Supplementary Figure 4) including Na_V_1.8 and Na_V_1.9, effects of HSTX-I on rNa_V_1.8 (rat Na_V_1.8) and mNa_V_1.9 (mouse Na_V_1.9) were investigated. As illustrated in **Figure 2**, HSTX-I reduced Na_V_1.8/1.9 currents with IC_50_ ∼2.44 ± 1.42 (**Figure 2C**) and 3.30 ± 2.48 (**Figure 2D**) μM, respectively. HSTX-I (3 μM) had no effect on G–V relationship and steady-state inactivation of rNa_V_1.8 (**Figure 2E**) channels. HSTX-I (3 μM) had no effect on steady-state inactivation of mNa_V_1.9 channels. In contrast, HSTX-I (3 μM) shifted steady-state activation to hyperpolarizability of ∼5 mV on mNa_V_1.9 (**Figure 2F**) channels.

Na_V_1.9 acts as a threshold channel that regulates excitability of DRG neurons to induce pain. Threshold depolarizing currents of small DRG neurons before and after application of HSTX- I (3 μM) were compared as illustrated in **Figures 2G,H**. Average current threshold of controls was 341.7 ± 15.37 pA (*n* = 6). After treatment with 3 μM HSTX-I, the current threshold was increased to 491.7 ± 27.13 pA (*n* = 6) which was significantly larger than that in the control (*P* = 0.0007). **Figure 2G** shows a small DRG neuron responding to subthreshold current injections (250–300 pA) and the threshold depolarizing current injections (350 pA) with graded membrane potential depolarizations before application of HSTX-I. After application of HSTX-I (3 μM), subthreshold current injections and threshold depolarizing current injections rose to 400–450 and 500 pA, respectively (**Figure 2H**).

### Analgesic Functions of HSTX-I in Rodent Models

**FIGURE 3:**
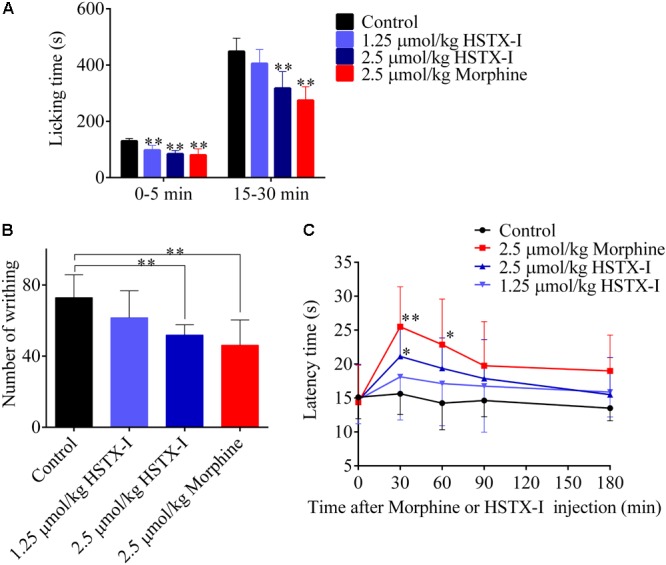
Analgesic activities of HSTX-I in rodent models. The analgesic activities of HSTX-I in mouse models of formalin-induced paw licking **(A)**, abdominal writhing induced by acetic acid **(B)** and hot plate test **(C)**, respectively. Data are represented as mean ± SEM, *n* = 6. HSTX-I and morphine were administrated by intraperitoneal injection. ^∗^*P* < 0.05 and ^∗∗^*P* < 0.01 are significantly different compared with the control (0.9% saline group).

Na_V_1.8/1.9 are preferentially expressed in nociceptors and have been showed to have a major role in pain (Swanwick et al., 2010; Dib-Hajj et al., 2015). Given its inhibitory effects on Na_V_1.8/1.9, HSTX-I likely exerts analgesic functions. As illustrated in **Figure 3A**, HSTX-I reduced the response of pain in both the first (neurogenic pain, 0–5 min) and the second phases (inflammatory pain, 15–30 min) in formalin-induced paw licking mouse model, suggesting that HSTX-I has similar effect on both neurogenic and inflammatory pain. In the first phase, licking time was reduced 25.4% (*P* < 0.01) and 36.5% (*P* < 0.01) by 1.25 and 2.5 μmol/kg HSTX-I, while in the second phase, the licking time was reduced 9.8 and 24.6% (*P* < 0.01), respectively. In the mouse model of abdominal writhing induced by acetic acid, 1.25 and 2.5 μmol/kg HSTX-I decreased writhing time by 15.5 and 28.8% (*P* < 0.01), respectively (**Figure 3B**). Hot plate model of mouse showed that the latency of pain response was delayed by intraperitoneal administration of HSTX-I. The latency was delayed 10.7 and 29% (*P* < 0.05) by 1.25 and 2.5 μmol/kg HSTX-I, respectively (**Figure 3C**). HSTX-I showed outstanding analgesic functions in several mouse models.

## Discussion

Voltage-gated sodium channels are integral transmembrane proteins that play a key role for the rapid depolarization of excitable cells (Hodgkin and Huxley, 1952). Their importance to pain perception is indisputable (Bagal et al., 2014). Activation of TTX-R sodium channels, especially Na_V_1.8, contributes to action potential electrogenesis in neurons (Akopian et al., 1996; Jarvis et al., 2007). Na_V_1.8 carries the majority of the current underlying the upstroke of the action potential in nociceptive neurons (Akopian et al., 1999; Renganathan et al., 2001). Recent genetic and functional findings indicate that Na_V_1.9 is linked to human pain disorder (Dennison et al., 2015). In order to successfully get blood meals and to anesthetize hosts, leeches possibly utilize salivary compounds acting on TTX-R sodium channels to inhibit activation of nociceptive neurons and overcome pain. Clinical experiments strongly indicate that leech therapy produces a strong and long lasting pain-reducing effect although no specific analgesic substance has been found.

The small peptide HSTX-I (ACKEYWECGAFLFCIEGICVPMI), which contains high density of half-cysteines as neurotoxins acting on ion channels, was identified and characterized from the leech salivary glands of *H. sylvestris* (**Figure 1**). It inhibited TTX-R Na^+^ channel currents and had no effect on voltage-gated Ca^2+^, K^+^, and TTX-S Na^+^ currents (Supplementary Figure 3). HSTX-I reduced Na_V_1.8/1.9 currents but only shifted steady-state activation of Na_V_1.9 to hyperpolarization (**Figure 2F**). This finding indicated the effects of HSXT-I on Na_V_1.8 and Na_V_1.9 were slightly different. HSTX-I may interact with different domains of Na_V_1.8 and Na_V_1.9. Further mutations experiments may give an answer. Due to its selective activity on Na_V_1.8 and Na_V_1.9, HSTX-I could be a valuable tool for the study of Na_V_1.8 and Na_V_1.9.

It is noted that Na_V_1.8 is cold resistant, meaning that it is essential for pain and in maintaining the excitability of nociceptors at low temperatures (Renganathan et al., 2001). HSTX-I possibly blocks excitability of host nociceptors at low temperatures, such as water environment where leeches’ generally inhabit. HSTX-I showed outstanding analgesic activity in several mouse models (**Figure 3**). It reduced the response of pain at both first (neurogenic pain, 0–5 min) and second phase (inflammatory pain, 15–30 min) with similar efficiency, suggesting that HSTX-I has similar effect on both neurogenic and inflammatory pain (**Figure 3A**). Na_V_1.8/1.9 may be safe targets for treatment of chronic pain because knocking out Na_V_1.8 or Na_V_1.9 in mice showed little adverse effects. HSTX-I selectively acted on Na_V_1.8/1.9 and showed significant analgesic effects in animal models, indicating it may be a promising lead molecule for development of analgesics.

## Conclusion

A novel peptide HSTX-I from *H. sylvestris* inhibited sodium channels (Na_V_1.8 and Na_V_1.9) and exerted direct analgesic function. The discovery of analgesic peptide HSTX-I in the leech riches our understanding of the molecular mechanisms of feeding strategies for hematophagia leeches. In addition, the current work is historically relevant because it reveals the specific analgesic substance and their pharmacological mechanisms in leech therapies.

## Author Contributions

RL, ZS, and XL designed the study and wrote the paper. GW and CL conducted most of the experiments, analyzed the results, and wrote the paper. WL and MZ conducted experiments on the purification of peptides. QL and PM conducted experiments on screening for ion channel function. CX and DL evaluated analgesic activity *in vivo*. QML and MR provided technical assistance and contributed to the preparation of the figures. All authors analyzed the results and approved the final version of the manuscript.

## Conflict of Interest Statement

The authors declare that the research was conducted in the absence of any commercial or financial relationships that could be construed as a potential conflict of interest.

## Footnotes

1 http://www.idtdna.com/Primerquest/Home/Index
