# Supplementary material for: Novel Sodium Channel Inhibitor From Leeches
Source: Front Pharmacol. 2018 Mar 6;9:186. doi: 10.3389/fphar.2018.00186 (PMC5845541; doi:10.3389/fphar.2018.00186)
Supplement: Supplementary file 1 [file Data_Sheet_1.doc]

Supplementary Materials

# Novel sodium channel inhibitor from leeches

Gan Wang ^1‡^, Chengbo Long ^1‡^, Weihui Liu ^1‡^, Cheng Xu ^1^, Min Zhang ^1,4^, Qiong Li ^2^, Qiumin Lu ^1,5^, Ping Meng ^1^, Dongsheng Li ^1^, Mingqiang Rong ^1,5^, Zhaohui Sun ^6*^, Xiaodong Luo ^2*^, Ren Lai ^1,3*^

*** Correspondence:** Corresponding Author: rlai@mail.kiz.ac.cn (RL), xdluo@mail.kib.ac.cn (XL) or zhaohui3@126.com (ZS)

# Supplementary Figures and Tables

## Supplementary Figure 1

**
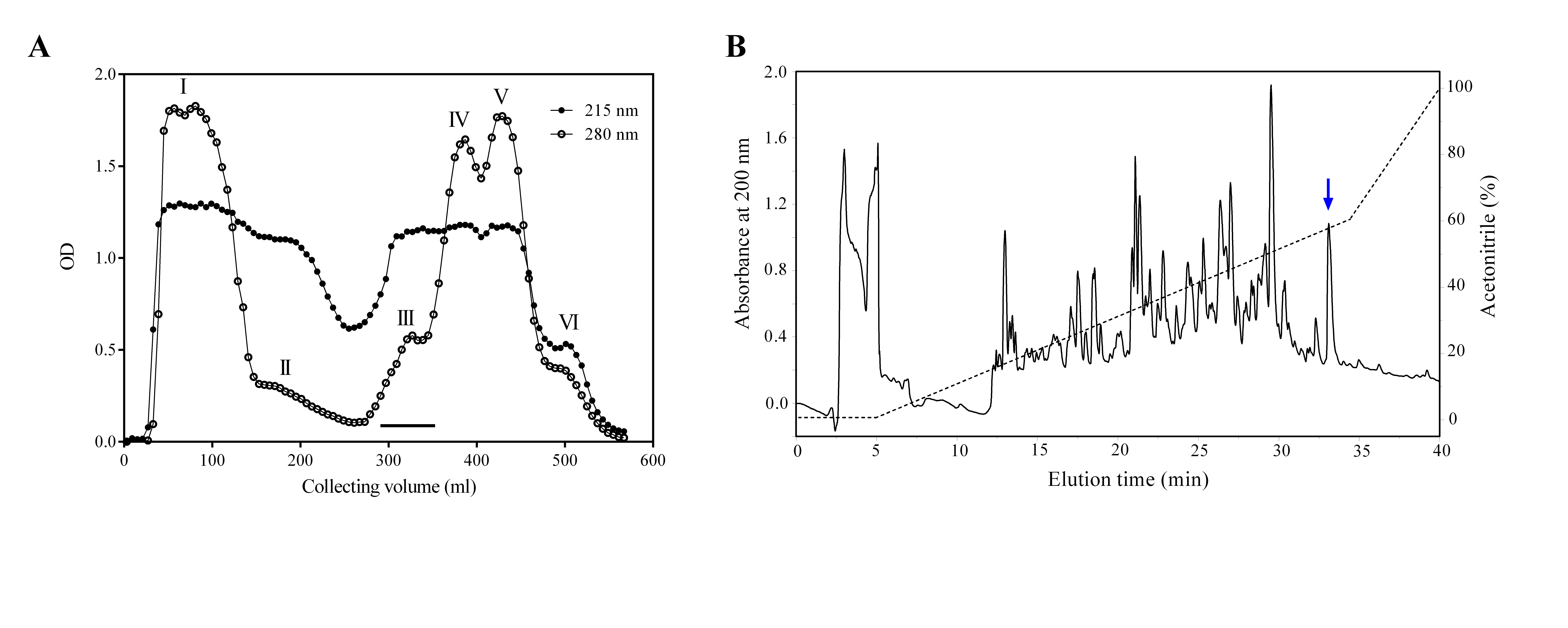
**

**Supplementary Figure 1.** **Identification of HSTX-I from *H. sylvestris saliva***. **(A)** SGE (500 mg) were divided into six peaks by G-50 gel filtration column according to absorbance at 280 nm. Peak III (indicated by a black bar) could inhibit TTX-R sodium channel currents. **(B)** Peak III in from **(A)** was further purified by a C_18_ reverse phase (RP) column and the peak with inhibitory effect on TTX-R sodium channels currents was indicated by a blue arrow. The other peaks in **(B)** did not have significant activity on Na_V_ channels.

## Supplementary Figure 2

**

**

**Supplementary Figure 2.** **Assignment of the Disulfide bonds of HSTX-I.** **(A)** The HPLC profiles of cycles of cysteine residues of 2, 8, 14, and 19, respectively after modification by iodoacetamide. The retention positions of alkylated cysteine residues are marked with black arrow. Cycle 8 and 19 have alkylated cysteine residue peaks, but cycle 2 and 14 have no. So, the assignment of the disulfide bonds of HSTX-I is Cys2-Cys14 and Cys8-Cys19. **(B)** MALDI-TOF-MS analysis of native HSTX-I under mono-isotopic model.

## Supplementary Figure 3

**

**

**Supplementary Figure 3.** **Effect of HSTX-I on voltage-gated Ca^2+^, K^+^ and Na^+^ channels.** The current traces of different voltage-gated channels before and after application of different concentrations of HSTX-I were recorded. **(A)** HSTX-I (3 μM) inhibited more than 68% of TTX-R Na^+^ channel current. **(B)**, **(C)**, and **(D)** HSTX-I had no effect on voltage-gated Ca^2+^, K^+^ and TTX-S Na^+^ channels.

## Supplementary Figure 4

**

**

**Supplementary Figure 4.** **HSTX-I inhibits TTX-R VGSCs on rat DRG neurons.** **(A)** Current–voltage relationship for TTX-R currents before and after application of HSTX-I. **(B)** Concentration-response curve for inhibition of TTX-R Na_V_ currents in DRG neurons by HSTX-I (n = 6) (holding potential -80 mV, stimulating voltage -10 mV). Inhibition of TTX-R currents was in a dose-dependent manner with an IC_50_ of ~1.316 ± 0.792 μM. Values are represented as mean ± SEM. **(C)** Effects of HSTX-I on voltage dependence of steady-state activation and inactivation of TTX-R VGSCs. HSTX-I at 0.75 and 1.5 μM induced -3.2 mV and -7.1 mV shift in the conductance-voltage relationship, respectively, but caused no significant shift in the voltage dependence of the steady-state inactivation of TTX-R DRG neurons.

## Supplementary Figure 5


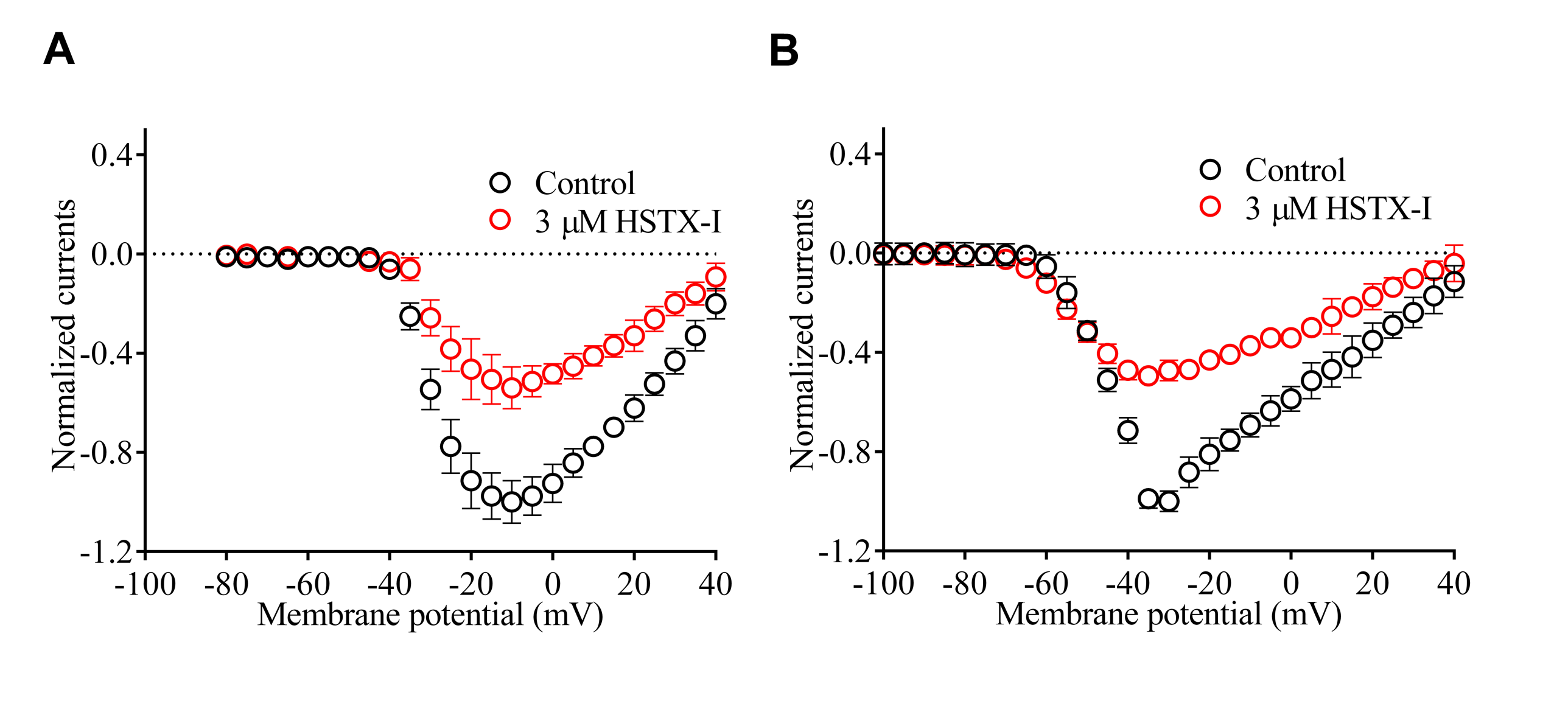


**Supplementary Figure 5.** **Current–voltage relationship for Na_V_1.8 and Na_V_1.9.** Current–voltage relationship for Na_V_1.8 **(A)** and Na_V_1.9 **(B)** currents before and after application of HSTX-I.

## Supplementary Figure 6


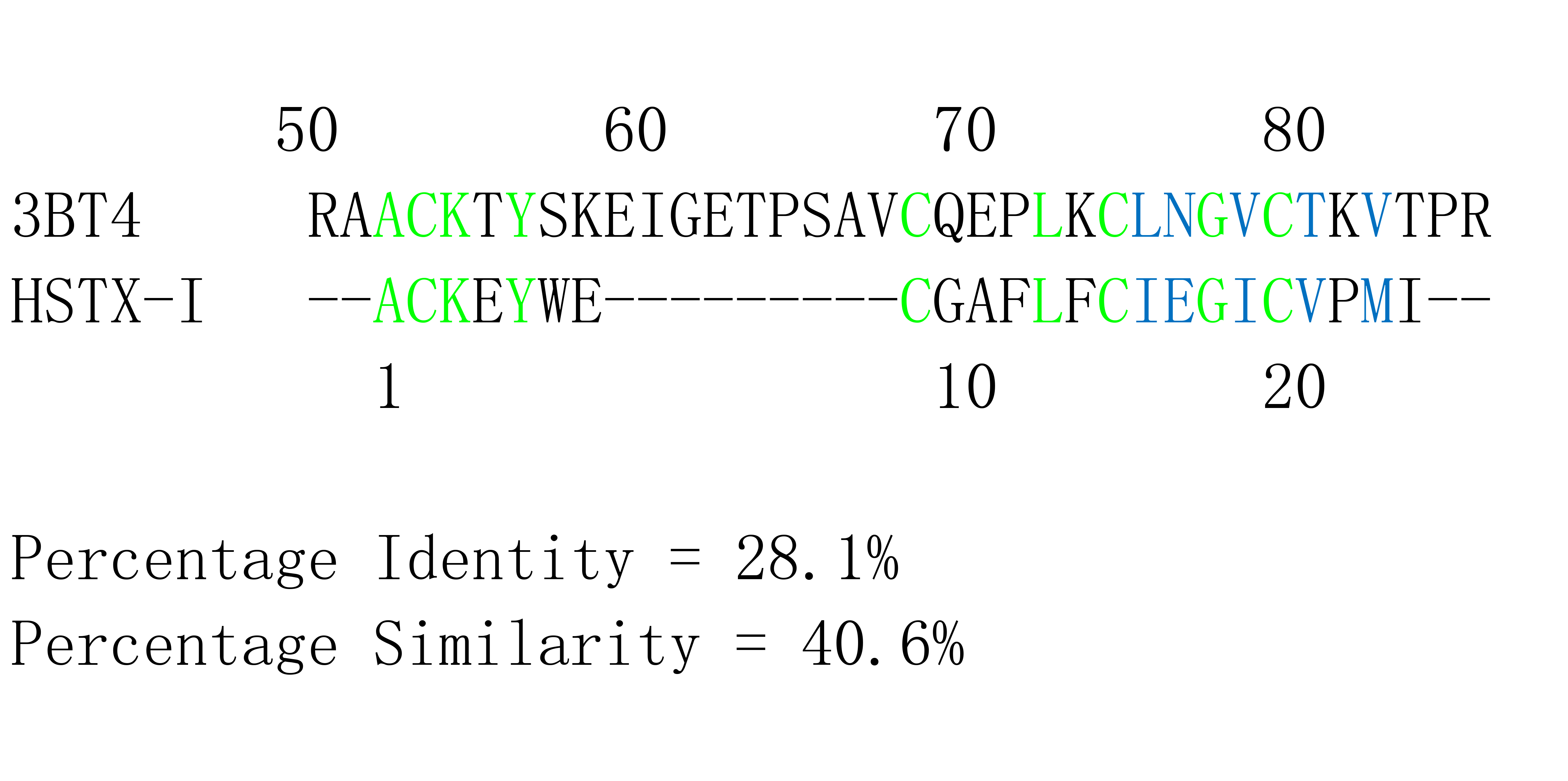


**Supplementary Figure 6.** **Sequence alignment between HSTX-1 and 3BT4 (PDB ID).** 3BT4 was used for the homology modeling of HSTX-I. Positions of amino acids in the sequence are indicated by numbers. The similar residues are shown in blue. The identical residues are highlighted in green. Hyphens indicate gaps.

## Supplementary Table

# Table S1, Information of the primers used for qPCR analysis

# qPCR primers

| Genes | Sequences (5’ to 3’) | Tm (℃) |
| --- | --- | --- |
| GAPDH | GAAGGTGAAGGTCGGAGTC | 58 |
|  | GAAGATGGTGATGGGATTTC |  |
| HSTX-I | CATCGGCAATGCTCAAGTTGAAGC | 60 |
|  | GAAACATTTTAATTTTTAGAGGTTCATCC |  |
